# Maternal Feeding Styles and Food Parenting Practices as Predictors of Longitudinal Changes in Weight Status in Hispanic Preschoolers from Low-Income Families

**DOI:** 10.1155/2016/7201082

**Published:** 2016-06-26

**Authors:** Sheryl O. Hughes, Thomas G. Power, Teresia M. O'Connor, Jennifer Orlet Fisher, Tzu-An Chen

**Affiliations:** ^1^USDA/ARS Children's Nutrition Research Center, Baylor College of Medicine, 1100 Bates, Houston, TX 77030, USA; ^2^Washington State University, 513 Johnson Tower, P.O. Box 644852, Pullman, WA 99164-4852, USA; ^3^Temple University, 1801 N. Broad Street, Philadelphia, PA 19122, USA

## Abstract

*Objective*. The aim was to investigate the influence of feeding styles and food parenting practices on low-income children's weight status over time.* Method*. Participants were 129 Latina parents and their Head Start children participating in a longitudinal study. Children were assessed at baseline (4 to 5 years old) and again eighteen months later. At each time point, parents completed questionnaires and height and weight measures were taken on the child.* Results*. The indulgent feeding style (parent-report at baseline) was associated with increased child BMI *z*-score eighteen months later compared to other feeding styles. Authoritative, authoritarian, and uninvolved feeding styles were not significantly associated with increased child BMI *z*-score. Child BMI *z*-score at Time 1 (strongest) and maternal acculturation were positive predictors of child BMI *z*-score at Time 2. Maternal use of restriction positively predicted and maternal monitoring negatively predicted Time 2 BMI *z*-score, but only when accounting for feeding styles.* Conclusion*. This is the first study to investigate the impact of feeding styles on child weight status over time. Results suggest that indulgent feeding predicts later increases in children's weight status. The interplay between feeding styles and food parenting practices in influencing child weight status needs to be further explored.

## 1. Introduction

Childhood obesity is associated with a host of negative health outcomes [1] resulting in a major public health concern for Americans [2]. It is well appreciated that environmental and behavioral factors contribute to childhood obesity with the immediate family having direct influences on its development [3]. Considerable evidence supports the premise that parents not only shape children's general development but also shape the development of child eating behaviors [4] and their weight status [5]. Studies linking parent-child behavioral processes to child weight have targeted parenting styles and parenting practices as playing a role in either fostering or preventing childhood obesity.* Parenting styles* are considered to be the stable overall attitude that parents have regarding how to socialize their children into becoming productive adults [6, 7]. In contrast,* parenting practices* are more goal oriented directives used to get the child to comply with a specific task [8]. In a recent review article, Shloim and colleagues [9] identified four studies (three longitudinal and one cross-sectional) that showed significant associations between parenting styles and child body mass index (BMI). For example, in one study children of parents with an indulgent parenting style were more likely to become overweight three years later compared to children of parents with an authoritative or authoritarian style [10]. In another study, high levels of protectiveness were associated with higher odds of children being overweight or obese five years later [11]. These longitudinal studies provide compelling evidence supporting the fact that some parenting styles contribute to the development of childhood obesity; however, problems exist in this literature due to inconsistent measurement of parenting and the lack of identification or examination of specific processes or mechanisms within these global parenting styles that either foster or thwart the development of appropriate child eating behaviors that lead to overweight and obesity.

*Feeding styles* also have been associated with child weight status mostly in cross-sectional studies. Feeding styles use a similar framework to parenting styles but specifically target parents' overall attitudes around the socialization of child eating behaviors [12]. Feeding styles are measured along two dimensions of demandingness and responsiveness specifically in the eating domain [12]. Demandingness refers to the level of demands parents make of their children during eating episodes, while responsiveness refers to how sensitive parents are to their child's individual needs during eating. High and low levels of these two dimensions translate into four feeding styles: authoritative parents (high demandingness/high responsiveness) make reasonable demands of their children while remaining sensitive to their child's needs; authoritarian parents (high demandingness/low responsiveness) are highly controlling and show little sensitivity to the child's needs; indulgent parents (low demandingness/high responsiveness) are sensitive to their child's desires during meals but provide little structure during eating allowing children extensive freedom; and uninvolved parents (low demandingness/low responsiveness) exhibit little control and involvement during eating.

The indulgent feeding style has been associated with higher child weight status across a series of cross-sectional studies with low-income families (see El-Behadli et al., 2015, for a review [13]). The indulgent feeding style has also been associated with child eating including self-selection of larger portion sizes and consumption [14], lower intake of fruit, vegetables, and dairy [15], and higher intake of energy dense foods [16]. Unlike studies linking general parenting styles to child weight status, studies examining feeding styles provide information regarding the specific mechanisms that foster the development of problematic eating behaviors in children. The premise is that parents who are indulgent are highly responsive to their child's eating preferences without setting appropriate limits. This does not help their child learn to pay attention to internal cues of hunger and satiety in our current food culture and instead fosters overeating and excessive intake of low nutrient, high calorie foods, thus contributing to child weight gain [17].

Certain food parenting* practices* have also been shown to be detrimental to the development of appropriate child eating behaviors. This includes restricting children's access to certain foods and pressuring the child to eat [5]. Much of this work has relied on the Child Feeding Questionnaire [18] to measure food parenting practices as it is the most common instrument used in the childhood obesity literature [5, 19]. The Child Feeding Questionnaire (CFQ) measures three food parenting practice constructs:* restriction* described as limiting child access to certain foods,* pressure to eat* described as making sure the child is eating enough, and* monitoring* described as keeping track of the child's intake of snacks and high fat foods. Restriction and pressure to eat are the only two feeding practice constructs that have been consistently associated with child weight status over multiple cross-sectional studies, with restriction positively associated with child weight status and pressure to eat showing a negative association [4, 5, 9, 19]. Data from longitudinal studies are less consistent. Although the Ventura and Birch [4] review found that restriction predicted weight gain in four of the five longitudinal studies they reviewed, Shloim and associates [9], in their review of studies since 2010, found that restriction positively predicted child weight status in only one of the four relevant longitudinal studies they located. Neither of the reviews identified longitudinal prediction of pressure to eat. More longitudinal studies need to be conducted with the CFQ to find some consensus regarding the causal link between these food parenting practices and child BMI—especially studies with more ethnically diverse samples at high risk for childhood obesity.

The overall aim of this study was to investigate the influence of feeding styles and food parenting practices on the weight status of low-income Hispanic preschool children over time. Hispanic families were chosen for this study as this ethnic group has a higher risk for childhood obesity relative to other ethnic groups [20]. Our primary goal was to examine indulgent feeding over time as indulgent feeding has consistently been linked to higher child weight in cross-sectional studies but not in a longitudinal design. An additional goal was to examine food parenting practices over time as previous studies have produced mixed results in associating these constructs to child weight longitudinally. We hypothesized that feeding styles, specifically the indulgent feeding style, would be associated with low-income child weight status over time. We expected that the influence of feeding styles would be beyond that of food parenting practices, because feeding styles assess parent interactions with their child in the context of both child- and parent-centered items. Food parenting practices only examine one construct at a time and do not capture other behaviors that parents exhibit when interacting with the child during meals (i.e., the global pattern of parent-child interactions). To provide the strongest test of the hypothesized associations, we controlled for a number of variables that have been associated with feeding behaviors in immigrant populations [21–25].

## 2. Methods

### 2.1. Participants

Participants were one hundred and twenty-nine Latina parents and their children participating in a longitudinal study (all mothers). Parents were recruited from Head Start centers in a large urban city in southeast United States when their child was 4 to 5 years old. One hundred and eighty-seven parents and their children participated at the first time point of the study. Eighteen months later, data from 144 parents and children were collected—129 had data on all of the variables for the present study.

### 2.2. Procedures

At Time 1, parents and their children came into our study laboratory on two separate days to participate in observational tasks related to self-regulation. On day two, parents completed a set of questionnaires while the child was involved in the tasks. Height and weight measures were taken on the child. All study staff were bilingual and parents were given the opportunity to complete the questionnaires in English or Spanish. About 77% of the parents preferred the Spanish questionnaires. At Time 2 (18 months later), parents and their children completed the same tasks as Time 1 over a two-day period. Height and weight measures were taken on the children. Participants were reimbursed for different aspects of their participation in the study with a possible total of $90 at Time 1 and $185 at Time 2. The study was reviewed and approved by the Institutional Review Board at the Baylor College of Medicine. The purpose of the study was explained to parents in English or Spanish and written consent was obtained before participation. Child verbal assent was secured as well. Parents were told that the purpose of the research was to study the development of children's eating behaviors.

### 2.3. Measures

All questionnaires used in this study have been translated into Spanish and back-translated into English to assure understanding of the wording and concepts. These questionnaires have been used successfully in previous studies with Hispanic participants [13].

#### 2.3.1. Demographics

Demographic information was obtained including birth dates (parent and child), ethnicity, gender, education, marital status, and immigrant status.

#### 2.3.2. Caregiver's Feeding Styles Questionnaire (CFSQ)

The CFSQ was used to assess feeding styles of parents in this study [12]. The CFSQ was designed specifically to assess feeding in low-income, ethnically diverse samples [12]. Seven child-centered and 12 parent-centered feeding directives are used to derive two dimensions of demandingness and responsiveness. Parents respond to the 19 directives on a 5-point Likert scale ranging from* never* to* always*. The dimension of responsiveness assesses promotion of child autonomy (e.g., reasoning, complimenting, and helping the child to eat), while controlling for overall feeding directives. The dimension of demandingness assesses the use of both child- and parent-centered directives. A cross-classification of high and low dimension scores identifies four feeding typologies: authoritative (high responsiveness, high demandingness), authoritarian (low responsiveness, high demandingness), indulgent (high responsiveness, low demandingness), and uninvolved (low responsiveness, low demandingness). Evidence of test-retest reliability, internal consistency, and convergent and predictive validity has been demonstrated [12]. The CFSQ has been validated with observations of parent/child interactions during dinnertime [17]. A more complete discussion of the scoring procedure can be found elsewhere [12].

#### 2.3.3. Child Feeding Questionnaire (CFQ)

The CFQ was used to assess food parenting practices in this study [18]. The CFQ measures four attitudes (perceived responsibility, perceived child weight, perceived parent weight, and concern about child weight) and three practices (restriction, pressure to eat, and monitoring). Only those subscales assessing food parenting practices were used in this study. These included restriction (e.g., I intentionally keep some foods out of my child's reach); pressure to eat (e.g., my child should always eat all the food on her plate); and monitoring (e.g., how much do you keep track of the high fat foods that your child eats?). This measure has been used and validated in low-income samples [12, 26].

#### 2.3.4. Children's Eating Behavior Questionnaire (CEBQ)

The CEBQ is a 35-item parent-report questionnaire measuring eight dimensions of child eating [27]. The eight dimensions include food responsiveness, emotional overeating, enjoyment of food, desire to drink, satiety responsiveness, slowness in eating, emotional undereating, and food fussiness. The factor structure, test-retest reliability, and internal consistency have been established [27]. The CEBQ has been used successfully in low-income samples [28]. To reduce the number of variables in the analyses, the three subscales related to the self-regulation of caloric intake—food responsiveness (e.g., my child is always asking for food), emotional overeating (e.g., my child eats more when worried), and satiety responsiveness (e.g., my child gets full before his/her meal is finished)—were used in this study as they have been linked to food parenting practices and child weight in Hispanic samples [29, 30].

#### 2.3.5. Bidimensional Acculturation Scale (BAS)

The BAS was used in this study to assess parents' acculturation into the US culture [31]. The BAS consists of three subscales: language use (e.g., how often do you speak English?), language proficiency (e.g., how well do you read in English?), and electronic media (e.g., how often do you watch television programs in English?). Four response categories are used for the language use and electronic media items (almost never, sometimes, often, and almost always). Four different response categories are used for the language proficiency items (very poorly, poorly, well, and very well). Per the developers' recommendations, two domains were created from the three subscales—a Spanish domain and an English domain [31]. Only the English domain was used in this study as almost 90% of the participants scored three or above on the response scale of one to four in the Spanish domain resulting in little variability.

#### 2.3.6. Anthropometrics

Height and weight measurements were taken on the child by trained staff members following a standard protocol to determine body mass index [32]. A stadiometer and an electronic self-calibrating digital scale were used to take the measurements. Children wore light clothing and were asked to remove their shoes. Measurements were recorded to the nearest 0.1 cm (height) and 0.1 kg (weight). Two height and weight measures were taken and averaged. Centers for Disease Control and Prevention Reference Standards were used to generate age- and gender-specific BMI* z*-scores [33]. Children were classified as normal weight (BMI ≤ 85th percentile), overweight (BMI > 85th ≤ 95th percentile), and obese (BMI > 95th percentile).

### 2.4. Statistical Analyses

Descriptives were run on all variables and examined to determine distributions and bivariate relationships (using Pearson or point-biserial correlation coefficients). The main study questions were tested with a hierarchical regression analysis. The dependent variable was the BMI *z*-score at the second time point. The independent variables were entered into the analysis in a sequence of blocks. To provide a stronger test of the association between variables, we strategically placed variables in certain blocks. For example, we statistically controlled for child eating behaviors before examining the role of parental behavior. This was done to address the concern that associations between parental feeding styles and food parenting practices may simply be responses to child eating behaviors. Furthermore, because many of the parents in the sample were immigrants to the USA, we controlled for acculturation in our analyses as well, since acculturation is often associated with child weight status and food parenting practices in immigrant samples [21–25].

Therefore, Block 1 included (a) BMI *z* at the first time point, (b) the demographic variables of child sex and age in months at the first time point, and (c) parental acculturation—English subscale of the acculturation questionnaire and whether the parent was born in the USA (dichotomous predictor). Block 2 included the three CEBQ child eating behavior subscales at the first time point (food responsiveness, emotional overeating, and satiety responsiveness) to control for child eating behaviors. Block 3 included food parenting practices—monitoring, pressure to eat, and restriction from the CFQ. Lastly, Block 4 included parental feeding styles from the CFSQ (one dichotomous predictor for each of three feeding styles—authoritarian, authoritative, and indulgent). For each dichotomous variable, the mother was assigned a “2” if she demonstrated a particular feeding style and a “1” if she did not. Only three feeding styles could be entered simultaneously into the regressions, because adding a fourth style (i.e., uninvolved) would provide no new information (if a mother had a “1” on all three feeding style variables, her style would be uninvolved). Indulgent, authoritarian, and authoritative feeding styles were chosen for entry into the equation because these three styles have most often been associated with child weight status (positively or negatively) in previous studies of general parenting or feeding styles [13]. To examine the relationship between the uninvolved style and weight change, an additional regression was run with the uninvolved style as the only feeding style predictor. All statistics were run using the Statistical Package for the Social Sciences (SPSS, Version 20.0, Chicago, IL). Statistical significance was set at *p* value < 0.05.

## 3. Results

### 3.1. Sample Characteristics

About an equal number of boys and girls participated. Most of the mothers were born outside of the United States, predominantly in Mexico. Fifty-six percent of the mothers were married and seventy-six percent did not work outside of the home. Sixty-three percent of the mothers had a high school education or less. Finally, ninety-seven percent of the children were born in the United States and fifty-one percent were overweight or obese. Sample characteristics are presented in Table 1.

Comparison of the 129 mothers and children whose data were analyzed here and the 58 parents and children in the Time 1 sample whose data were not analyzed for the present paper (either because they dropped out of the study or they had missing data on acculturation) revealed only one significant difference in the variables in Table 1. Mothers in the current study were more likely to have been born outside of the USA (84%) than parents whose data were not analyzed here (66%): *χ* ^2^(1) = 8.57, *p* < 0.01.

### 3.2. Bivariate Correlations

Presented in Table 2 are the correlations between all study variables. BMI *z* at both time points was positively correlated with indulgent feeding. In contrast, BMI *z* was negatively correlated with pressure to eat (both time points) and the authoritarian feeding style (Time 2).

### 3.3. Hierarchical Block Regression

The results of the hierarchical block regression are presented in Table 3. Table 3 displays the standardized regression coefficients (*β*) and the adjusted squared multiple correlation (adjusted *R* ^2^) for each step. In all of the analyses, BMI *z* at the first time point was positively associated with BMI *z* at the second time point and was the strongest predictor (standardized *β* ranged from 0.915 to 0.929, *p* < 0.001). This was followed by the English subscale of the acculturation questionnaire which was positively related with BMI *z* (standardized *β* ranged from 0.094 to 0.108, *p* < 0.05) across all four steps. After Step 3 (addition of food parenting practices), the *R* ^2^ change was not significant with adjusted *R* ^2^ = 0.857 and *F*(11,117) = 70.649. With all 14 independent variables included in the model (Step 4), adjusted *R* ^2^ = 0.867, *F*(14,114) = 60.523, *R* ^2^ change of 0.010 satisfied *F* change (3,114) = 3.93, and *p* < 0.01, indicating the addition of the feeding styles from the CFSQ significantly improved *R* ^2^. In addition to BMI *z* at Time 1 and English acculturation, food parenting practices of monitoring (*β* = −0.068, *p* = 0.046) and restriction (*β* = 0.082, *p* = 0.04) and the indulgent feeding style (*β* = 0.114, *p* = 0.045) were significant predictors of BMI *z* at Time 2 in the full model (Block 4). Restriction and indulgent feeding style were positive predictors of BMI *z* at Time 2; monitoring was a negative predictor. A separate model with the uninvolved style as the only feeding style predictor (including all of the other predictors) showed no significant effect for uninvolved feeding style: *β* = −0.02, *p* = 0.52.

## 4. Discussion

This study is the first to investigate the impact of feeding styles on children's weight status over time and did so among a sample of low-income Hispanic children from Head Start programs. In support of the hypothesis, indulgent feeding style assessed when the child was an average of 4.8 years old and was associated with increased BMI *z*-score 18 months later. Authoritative, authoritarian, and uninvolved feeding styles were not associated with changes in BMI *z*-score over time. The relationship of an indulgent feeding style to child BMI *z*-scores made a significant contribution to explaining increases in the child's BMI *z*-score over and beyond the child's baseline BMI *z*-score, demographics, and the child's eating behaviors at 4 years of age. Other significant predictors of time to BMI *z*-score in the final model were Time 1 child BMI *z*-score (strongest predictor), parent's level of acculturation, and parent's use of restriction and monitoring food parenting practices. While the former two were risk factors for greater BMI *z*-score at time two (positive associations), monitoring at time one was protective or negatively associated with the child's BMI *z*-score over time.

It is noteworthy that it was the indulgent feeding style that was associated with child's BMI *z*-score over time since the indulgent feeding style has consistently been associated with higher child BMI *z*-score or percentile in cross-sectional studies assessing the same construct among low-income African American and Hispanic [12, 28, 34], rural African American, Hispanic, and White [35] and recent immigrant samples [36]. The indulgent feeding style results when parents make few demands on their children during feeding and use mostly child-centered feeding directives. This relatively permissive style of feeding does not provide children with the same level of scaffolding, limit setting, and rules seen among parents who use authoritative or authoritarian feeding styles. Previous work among low-income families found that indulgent and uninvolved feeding styles were associated with lower intake of fruit, vegetables, and dairy [15], providing one possible mechanism through which feeding styles impact child weight status over time. However, it is apparent in the analysis presented here that the indulgent feeding style puts children at greater risk for excessive weight gain over time. This suggests that other mechanisms are also involved. It has been hypothesized that parents who are indulgent may use food to show love and affection for their child, which may contribute to greater energy intake and be an additional mechanism by which indulgent feeding styles contribute to children's excess weight gain [34]. Moreover, the indulgent feeding style may interfere with children's self-regulation of caloric intake because low levels of parental limit setting and use of rules may lead to children overeating and ignoring their internal satiety cues [17]. One strength of the feeding style construct is that it assesses parenting influences on child eating in context of both child- and parent-centered directives, giving a more global assessment of how parents interact with their child during meals compared to food parenting practices.

In this study, the positive association of restrictive food parenting practices and the negative association of monitoring food parenting practices only contributed to the child's BMI *z*-score at Time 2, when also controlling for the feeding styles. It is possible that food parenting practices are expressed differently among different feeding styles, and therefore both need to be considered when assessing children's weight gain over time. In fact, restriction and monitoring have had more mixed results in studies assessing their association with children's weight or BMI, as compared to feeding styles [9]. Most of the cross-sectional studies have supported a positive association with restriction and child BMI, but longitudinal findings have been equivocal [4, 9]. Monitoring, on the other hand, has generally not been linked with child BMI in cross-sectional nor longitudinal studies [9] but was linked to better dietary quality two years later [37]. One small study previously associated monitoring at age 5 with reduced child BMI *z*-score at age 7 among low obesity risk children, but not high obesity risk children, as determined by the parent's weight status [38]. Therefore, researchers and health care providers need to take both food parenting practices and feeding styles of parents into consideration when intervening and treating childhood obesity.

It has been argued that much of the association of feeding styles and feeding practices with children's weight status is due to the parenting behaviors being in response to their child's eating behaviors, which may actually drive the association [39]. In the analysis presented here, three child eating behavior characteristics (food responsiveness, emotional overeating, and satiety responsiveness) previously linked to either parent food parenting practices or child weight status in cross-sectional studies [29, 30] were assessed. While satiety responsiveness was significantly negatively correlated with the child's BMI *z*-score at Time 1, none of these child eating behaviors correlated with the BMI *z*-score at Time 2. Nor did any contribute to changes in the child's BMI *z*-score over time when controlling for demographics and acculturation or when considering feeding styles and food parenting practices.

Acculturation, or how much an immigrant adapts to the new culture in which they reside, has been positively associated with the risk of childhood obesity [21–23, 25]. We have previously shown that the level of acculturation of Hispanic parents from this sample at baseline was associated with lower use of restrictive practices, while parents born in the USA were more likely to report an indulgent feeding style and less likely to report an authoritarian feeding style [24]. Here we demonstrate that the parent's level of acculturation to the English/American culture as assessed by English language usage, English language proficiency, and use of English media was predictive of increased child BMI *z*-score over time. These results are consistent with other studies showing that more acculturated Hispanics tend to have higher levels of obesity than recent immigrants [21, 25], possibly a result of greater exposure to the obesogenic environment in the USA [40, 41].

The limitations of this study should be acknowledged. This study included a convenience sample of Hispanic Americans in one city in southwestern USA and may not generalize to others. The sample size is relatively small, and we did not have complete data on all participants that started the study. However, the only demographic difference between those that provided baseline data and those that completed the study was parental birthplace. The feeding styles and food parenting practices were assessed by self-report. Self-report instruments are more likely than other objective assessments of parenting (e.g., observations) to have errors introduced, such as social desirability biases and biases associated with self-awareness. However, the CFSQ has been validated by observations in the home, providing support that this self-report instrument is capturing important differences in how a parent interacts with their child during a family meal in their home [17]. In addition, we used the feeding style and food parenting practice instruments that have most commonly been used in studies linking parenting to child BMI [5, 19], which allows for better comparison across studies. Finally, we used a *p* value of *p* < 0.05 and did not correct for Type I error. Given the large number of parameters in the final regression for the number of participants, it is important to replicate these findings in future research.

## 5. Conclusion

An indulgent feeding style was linked with increases in children's BMI *z*-score from 4.8 years of age to 18 months later, providing additional support of the importance of feeding styles in influencing child weight status. By controlling for the child's eating behaviors at baseline, we provide support that feeding styles influence child eating behavior and are not just a covariate in reaction to the child's eating behavior characteristics which impact weight. Our results suggest that there may be interplay between food parenting practices and feeding styles in influencing the child's weight status over time. This needs to be investigated further.

## Figures and Tables

**Table 1:** Characteristics of the sample at the first time point (*n* = 129).

Parent sex, female	100.0%
Child sex, female	45.0%
Parent age, mean in years (SD)^a^	32.01 (6.68)^a^
Child age, mean in years (SD)^a^	4.78 (0.46)^a^
Education of parent	
High school diploma or less	62.8%
Some college or more	37.2%
Employment status, currently employed	24.0%
Marital status	
Married	55.8%
Never married	13.2%
Widowed, separated, or divorced	31.0%
Parent immigrant status	
Born in the USA	15.5%
Born in Mexico	64.3%
Born in Central America	20.2%
Child immigrant status	
Born in the USA	97%
Child BMI categories	
Normal (<85th percentile)	48.8%
Overweight (85th to <95th percentile)	21.7%
Obese (≥95th percentile)	29.5%

^a^Standard deviation.

**Table 2:** Correlations between study variables.

	1	2	3	4	5	6	7	8	9	10	11	12	13	14	15
(1) Female	—														
(2) Age in months	0.03	—													
(3) Food responsiveness	−0.31^*∗∗*^	−0.01	—												
(4) Emotional overeating	−0.22^*∗*^	0.08	0.62^*∗∗*^	—											
(5) Satiety responsiveness	0.10	0.11	−0.35^*∗∗*^	−0.04	—										
(6) Acculturation	0.04	0.10	−0.03	−0.05	0.08	—									
(7) Born in USA	0.09	0.04	−0.08	−0.09	0.13	0.59^*∗∗*^	—								
(8) Monitoring	0.08	−0.04	−0.08	−0.11	−0.02	0.10	0.08	—							
(9) Pressure to eat	−0.18^*∗*^	−0.12	0.07	−0.14	−0.05	−0.26^*∗∗*^	−0.31^*∗∗*^	0.09	—						
(10) Restriction	−0.08	0.05	0.17^*∗*^	0.18^*∗*^	0.05	−0.20^*∗*^	−0.34^*∗∗*^	0.04	0.30^*∗∗*^	—					
(11) Authoritarian	0.02	0.11	0.01	0.14	0.23^*∗∗*^	−0.18^*∗*^	−0.21^*∗*^	−0.18^*∗*^	0.26^*∗∗*^	0.34^*∗∗*^	—				
(12) Indulgent	0.09	0.03	0.01	−0.20^*∗*^	−0.15^*∗*^	0.21^*∗*^	0.26^*∗∗*^	0.07	−0.14	−0.42^*∗∗*^	−0.54^*∗∗*^	—			
(13) Authoritative	0.00	−0.05	0.11	0.14	−0.09	0.08	−0.02	0.13	−0.12	0.01	−0.36^*∗∗*^	−0.31^*∗∗*^	—		
(14) BMI *z* (Time 1)	−0.05	0.14	0.14	0.06	−0.20^*∗*^	0.07	0.01	−0.03	−0.21^*∗*^	−0.06	−0.16	0.17^*∗*^	0.02	—	
(15) BMI *z* (Time 2)	−0.04	0.14	0.13	0.06	−0.15	0.16	0.06	−0.06	−0.20^*∗*^	−0.04	−0.22^*∗*^	0.24^*∗∗*^	0.03	0.92^*∗∗*^	—

^*∗*^ *p* < 0.05;  ^*∗∗*^ *p* < 0.01.

**Table 3:** Regression analysis predicting child BMI *z* at Time 2 (*N* = 129).

	Block 1	Block 2	Block 3	Block 4
Model adjusted *R* ^2^	0.859	0.857	0.857	0.867
*F* (model)	*F* (5,123) = 157.288^*∗∗∗*^	*F* (8,120) = 96.818^*∗∗∗*^	*F* (11,117) = 70.649^*∗∗∗*^	*F* (14,144) = 60.523^*∗∗∗*^

*F* (*R* ^2^ change)		*F* (3,120) = 0.328	*F* (3,117) = 0.982	*F* (3,114) = 3.930^*∗∗*^

Independent variables	Std beta	Std beta	Std beta	Std beta

Child sex (ref group: male)	0.008	0.011	0.018	0.012
Child age in months	0.001	−0.004	−0.006	−0.005
Child BMI *z* (Time 1)	0.919^*∗∗∗*^	0.924^*∗∗∗*^	0.929^*∗∗∗*^	0.915^*∗∗∗*^
Acculturation (English domain)	0.103^*∗*^	0.102^*∗*^	0.108^*∗*^	0.094^*∗*^
Born in USA	0.013	0.015	−0.003	0.003
Food responsiveness		0.016	0.004	−0.027
Emotional overeating		0.006	0.007	0.038
Satiety responsiveness		0.034	0.026	0.044
Monitoring			−0.048	−0.068^*∗*^
Pressure to eat			0.021	0.038
Restriction			0.034	0.082^*∗*^
Authoritarian^a^				−0.033
Authoritative^a^				0.050
Indulgent^a^				0.114^*∗*^

^*∗*^ *p* < 0.05; ^*∗∗*^ *p* < 0.01; ^*∗∗∗*^ *p* < 0.001.

Std beta: standardized beta coefficient.

^a^For each feeding style variable, a dichotomous predictor was used with a “2” assigned to mothers who showed that feeding style and a “1” to those who did not.
